# Identification and Analysis of Critical Suicide Sites and Factors in Castilla-La Mancha (2020–2024): Forensic and Healthcare Collaboration for Prevention

**DOI:** 10.3390/bs16010007

**Published:** 2025-12-19

**Authors:** Beatriz Vallejo-Sánchez, Natalia Solano-Pinto, Ana Huertes-Del Arco, Valeriano Muñoz, Mónica Casillas, Carolina Arroyo, Fernando Moreno

**Affiliations:** 1 Central Services of the Castilla-La Mancha Health Service (SESCAM), 45007 Toledo, Spain; 2 Health, Education and Society Research Group, Health Research Institute of Castilla La Mancha (IDISCAM), 45071 Toledo, Spain; natalia.solano@uclm.es; 3 Faculty of Languages and Education, Nebrija University, 28043 Madrid, Spain; 4 School of Health Sciences and Education, Open University of Madrid (UDIMA), 28400 Madrid, Spain; ana.huertes@udima.es; 5 Department of Psychology, University of Castilla La Mancha, 45071 Toledo, Spain; 6 Department of Personality, Assessment and Psychological Treatments, Faculty of Psychology, National University of Distance Education (UNED), 28040 Madrid, Spain; 7 Forensic Pathology Section, Institute of Legal Medicine and Forensic Sciences of Toledo, 45003 Toledo, Spain; valeriano.munoz@justicia.es; 8 Pathology Service, Institute of Legal Medicine and Forensic Sciences of Albacete, Cuenca, and Guadalajara, 02001 Albacete, Spain; monica.casillas@justicia.es; 9 Institute of Legal Medicine and Forensic Sciences of Toledo, 45003 Toledo, Spain; carolina.arroyo@justicia.es; 10 Clinical Service, Institute of Legal Medicine and Forensic Sciences of Albacete, Cuenca, and Guadalajara, 02001 Albacete, Spain; fernando.moreno@justicia.es

**Keywords:** suicide prevention and control, risk factors, forensic medicine, epidemiological surveillance, public health, descriptive epidemiology

## Abstract

Suicide is a major public health concern worldwide, and identifying the spatial patterns associated with its occurrence is essential for designing effective preventive strategies. This study aimed to identify and characterize suicide locations in two provinces of Castilla-La Mancha, Spain, using a descriptive and retrospective analysis of 421 cases recorded by the Institutes of Legal Medicine and Forensic Sciences of Toledo and Albacete between 2020 and 2024. Locations were classified as critical or non-critical based on recurrence and public accessibility, and logistic regression was used to explore predictors of suicide in public settings. Results showed that 82% of cases involved men, yielding a 5:1 male-to-female ratio that exceeds the national average; the mean age was 56.6 years, and hanging was the most frequent method (56.1%). Most suicides occurred in private environments, and only one location met the criteria for a critical site. These findings indicate that spatial clustering plays a minimal role in the regional suicide burden and that prevention efforts should prioritize means restriction and early detection in private settings, along with broader measures for dispersed public cases rather than hotspot-focused interventions. The study underscores the importance of systematically incorporating spatial information into forensic records to improve regional suicide surveillance and inform more targeted, context-sensitive prevention policies.

## 1. Introduction

Suicide is one of the leading causes of preventable death worldwide, with more than 700,000 deaths annually (World Health Organization, 2025). Within this global phenomenon, there is a particular subset of deaths that occur in specific public places known as “hotspots,” or locations with a high incidence of suicide. These are defined as locations that have a disproportionate number of suicides compared to other environments (Joyner et al., 2025). Some authors establish as a criterion the location’s having more than one suicide or serious attempt documented in a five-year period, along with additional conditions such as public or semi-public access, availability of a highly lethal method, relative isolation that would hinder early intervention, or local recognition as a recurring location for suicides (Owens et al., 2015; Pirkis et al., 2015; Too et al., 2024). Other authors, however, adopt different criteria for suicide hotspots, such as having a rate of at least 0.5 suicides per year over a decade (Hemmer et al., 2017). Critical sites often include infrastructure such as bridges, cliffs, railways, subway stations, tall buildings, or coastal areas, where the method used frequently results in death. The choice of these locations may be motivated by their reputation, personal significance, or association with previous deaths, even when people must travel from distant areas (Joyner et al., 2025).

The relevance of public locations is notable due to the high social, emotional, and media impact they generate. These events are often more visible to the community, affecting witnesses, family members, and emergency personnel (Giupponi et al., 2019; National Institute for Mental Health in England, 2006), and can trigger an imitation or contagion effect (known as the “Werther effect”), especially when the media reports in detail on the location or characteristics of the critical site (Marzano et al., 2025). In addition to the human consequences, these episodes have economic, legal, and logistical repercussions for the institutions responsible (Jain et al., 2024).

According to Merli and Costanza (2024), the percentage of suicides occurring at such locations varies with the context. For example, jumping from a great height accounts for fewer than 10% of total suicides, although this figure can increase substantially in places with easy access. Studies conducted at specific locations in New York found that the percentage increased to 24%; in Hong Kong, to 45%; in Singapore, to 60%; and in San Francisco, to 70%. Recently, Shin et al. (2025), in an analysis of 42,656 suicide cases, noted that 25.2% had occurred in public places. 

Prevention strategies at critical sites have been based on four strategic axes (Cox et al., 2013; Joyner et al., 2025; Owens et al., 2009): (a) restricting access to lethal means through physical barriers that block access to the critical site, nets to cushion and/or prevent falls, or platform doors that open only to allow access to trains; (b) facilitating third-party intervention through surveillance, patrols, or security cameras; (c) encouraging people to seek help through crisis hotlines or preventive signage that may deter suicidal intent; and (d) responsible communication, promoting a responsible change in narrative in the coverage of suicide.

Scientific evidence supports the effectiveness of these interventions. Systematic review studies and meta-analyses (LeFevre, 2014; Too et al., 2025) indicate that measures to restrict access to lethal methods can reduce suicide deaths by up to 91–93%; in certain cases, implementing barriers has even eliminated cases entirely. These figures are supported by confirmation that people who wish to die by suicide but encounter restrictions at their chosen location do not repeat the attempt elsewhere (Cox et al., 2013; Pirkis et al., 2015; Too et al., 2025). An example of a critical site in the United States is the Golden Gate Bridge in San Francisco, where the installation of preventive nets reduced suicides by more than 80% (Mackenzie et al., 2016). At elevated sites in Switzerland, the placement of barriers achieved a 69% reduction and that of nets a 77% reduction (Hemmer et al., 2017). In Asia, doors at train platforms have been shown to reduce suicides by 76% in Japan (Ueda et al., 2015), 89% in South Korea (Chung et al., 2016), and 91% in China (Xing et al., 2019).

Consistent with this evidence, a recent umbrella review (Nevarez-Flores et al., 2025) that integrated 12 international systematic reviews concluded that means restriction strategies are an empirically proven and effective measure for suicide prevention globally. The results indicate that the greatest effectiveness is achieved when interventions are adapted to the most prevalent methods of suicide and the local sociocultural context, minimizing the likelihood of method substitution.

Likewise, significant decreases of 50–61% have been observed through the installation of signage promoting help, and decreases of 47% have followed interventions that increase the likelihood of detection and intervention by third parties (Pirkis et al., 2015).

Furthermore, economic cost-effectiveness assessments of these measures have found them to be profitable investments. Bandara et al. (2022) estimated that installing barriers on bridges in Australia yielded a return of US $2.40 for every US dollar invested over a ten-year period. The evidence was less consistent in the case of cliffs, reinforcing the need for further research in this area. 

In Spain, the first Suicide Prevention Action Plan was published in 2025 (Comisionado de Salud Mental, Ministerio de Sanidad, 2025). It considers key preventive strategies to include intensifying measures that limit access to lethal methods, identifying and acting on “critical sites,” and improving the design of public spaces to increase safety, along with other measures already initiated years ago, such as strengthening control over the acquisition of toxic substances and the possession of weapons (in collaboration with the Spanish Medicines Ag2015 is changedency and the Ministry of the Interior). However, to date, there are no national publications analyzing the cost-effectiveness of such measures, despite the fact that some locations are socially recognized for their high incidence of suicide. One possible reason may be the scarcity of data on the exact locations of suicide deaths, a relevant factor for social and health policy planning. 

In this context, based on the relevance of studying “hotspots” and the lack of available data for the strategic planning of preventive actions, this study was carried out within the framework of a collaboration between the Castilla-La Mancha Health Service and the Institutes of Legal Medicine and Forensic Sciences (IMLCF), as well as several universities in the region and neighboring areas. The primary objective is to analyze suicides recorded between 2020 and 2024 in the provinces of Toledo and Albacete in order to ascertain the geographical distribution of critical sites, whether concentrated in public places (such as elevated infrastructure, natural environments, or transportation areas in Spain) or in private spaces, and to reveal associated sociodemographic characteristics that allow for the adoption of specific prevention strategies. The secondary objective is to identify possible critical sites and propose preventive measures adapted to the local contexts, based on available scientific evidence.

## 2. Materials and Methods

### 2.1. Study Design

This observational, descriptive, and retrospective study examines suicides that occurred in two provinces of the Autonomous Community of Castilla-La Mancha, Toledo and Albacete, during the period spanning 2020 to 2024. Data collection was carried out by IMLCF professionals in these provinces through the systematic analysis of records and the study of individual case files, allowing for a comprehensive reconstruction of each event.

### 2.2. Participants and Scope

The data available in the forensic archives of the IMLCF of Toledo and Albacete, collected during the medico-legal investigations of deaths by suicide between 2020 and 2024, were analyzed. Data from the provinces of Guadalajara, Ciudad Real, and Cuenca were not considered because of diminished accessibility due to differences in registration.

### 2.3. Study Variables

For each case identified, the following variables were recorded: date of the event (day, month, year); municipality and province; age (in years); sex (male, female, other); place of death; method used; prevention method suggested as likely to be effective (based on the suggestions provided by Too et al., 2025); and other relevant circumstances (qualitative and descriptive information collected to facilitate the identification and classification of locations).

In the case of places of death, data collection used a coded classification based on the situations most frequently reported in the literature, with researchers providing qualitative information that allowed for the identification of high-incidence sites (such as place names). The categories for this variable were: 1. bridge; 2. cliff; 3. tall public building (upper floors or roof); 4. train/subway station or railroad tracks at a location other than a station (differentiated in observations); 5. field; 6. forest; 7. road or path; 8. river/reservoir/lake; 9. public building (interior); 10. other; 11. place not classifiable as a “hotspot” due to being a private space or residence (with observations differentiating between residences, workplaces, and jumps from a residence to a public road). In relation to this last category, to facilitate the researchers´ classification task, several additional criteria were provided for those places that, in contrast, would be “classifiable as critical sites” (Owens et al., 2015; Pirkis et al., 2015; Too et al., 2024), specifically: 1. having public or semi-public access (as opposed to a private residence or workplace); 2. offering access to a highly lethal method (e.g., bridges or other elevated infrastructure, railroad tracks, deep bodies of water, cliffs, or high-speed roads); and 3. being sufficiently isolated to hinder early intervention. 

In a second step, after this initial classification, those places “classifiable as critical sites” were considered actual critical sites only if they met the most common definition found in the literature (Bandara et al., 2022), which is those places where more than one suicide had been documented within a 5-year reference period.

Classification of methods chosen followed a standardized typology based on the behaviors most frequently used to die by suicide, namely: 1. jumping from a height; 2. hanging/strangulation/suffocation; 3. firearm; 4. poisoning by drugs or chemicals; 5. inhalation of carbon monoxide or gases; 6. drowning; 7. burns/self-immolation; 8. cutting or sharp weapon; 9. collision with a moving vehicle; 10. electrocution; 11. other.

### 2.4. Data Analysis

Descriptive analyses were performed to obtain the mean and range of the continuous quantitative variable in the study (age), as well as the absolute and relative frequencies (percentages) of the nominal qualitative variables: sex, province, classification of the location as a critical site, location, and method. The descriptive analysis was both performed for the total sample and stratified by province (Toledo/Albacete) and by type of site (classifiable as critical or not).

For the analysis of association between variables, the age variable was recoded according to age ranges based on the minimum and maximum values observed. The following categories were defined: adolescence (15–17 years), emerging adulthood (18–29 years), young adulthood (30–44 years), middle adulthood (45–59 years), older adulthood (60–74 years), and old age (≥75 years). 

## 3. Results

The analysis included 421 cases of suicide recorded from 2020 through 2024, with a mean age of 56.68 years (SD = 18.34), and a range between 15 and 97 years. Men accounted for 82.4% of cases, and more than half (64.4%) of the cases occurred in the province of Toledo. In both Toledo and Albacete, a higher proportion of men than of women was observed (Toledo: 84.5%; Albacete: 78.7%). The predominant age group was middle adulthood (45–59 years), with 34.7% in Toledo and 38.7% in Albacete.

The most frequent location, chosen in 9% of cases, was the countryside, followed by high places (bridges, cliffs, tall buildings), which accounted for 8.2%, and public buildings, at 4.8%.

As for method, hanging was the most frequent (56.1%), followed by jumping (16.6%) and ingesting drugs or chemicals (9.7%).

The years with the fewest cases were 2020 (76 cases) and 2024 (78 cases), while the highest numbers were recorded in 2022 (93 cases) and 2023 (89 cases). In seasonal terms, 56.1% of cases in Toledo and 61.4% in Albacete occurred during spring and summer.

Overall, 74.3% of the suicides analyzed occurred at public locations, and with regard to locations that could possibly be classified as critical sites, there were only two cases of location coincidence, i.e., of two or more cases occurring at the same location during the five-year period analyzed. Therefore, only one critical site was found during this period, specifically in the province of Toledo.

Table 1 presents the data cross-tabulated between the variables of location and method, using slashes (/) to differentiate between the provinces of Toledo and Albacete.

Of the 108 cases that occurred in public places, 10.2% (11 cases) involved women, with the province of Toledo being more highly represented (at 67.6%) than Albacete, where the preponderance of cases occurred in the private sphere.

**Figure 1:**
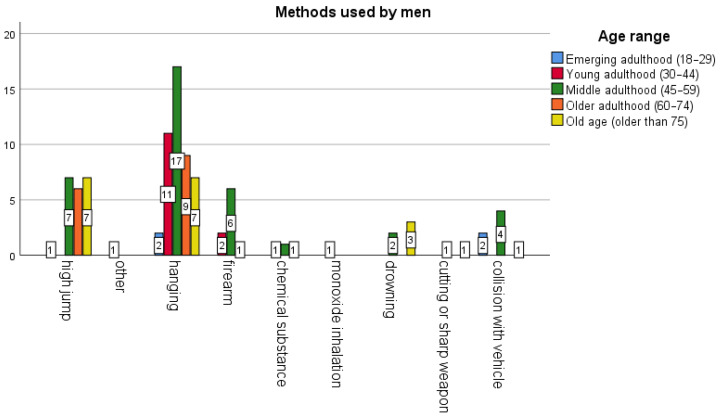
Number of cases involving men (n = 97) at public locations classifiable as critical sites. Analysis by age range and method.

**Figure 2:**
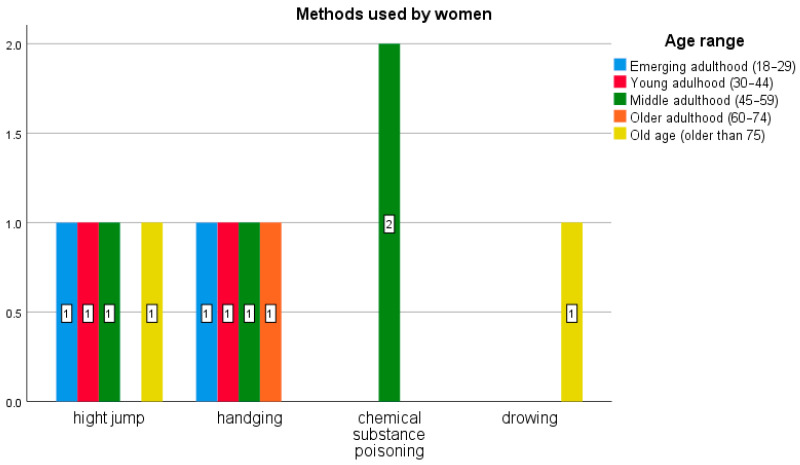
Number of cases involving women (n = 11) at public locations classifiable as critical sites. Analysis by age range and method.

Figure 1 and Figure 2 show that the predominant age group was middle adulthood (45–59 years) for both men and women. The most commonly used method for men was hanging (46 cases), followed by jumping from a height (21) and firearms (9). For women, it was jumping from a height (4), hanging (4), then poisoning (2) and drowning (1). Thus, the method used varied by sex: women did not use collision with a vehicle, a knife, a firearm, or inhalation of monoxide.

**Figure 3:**
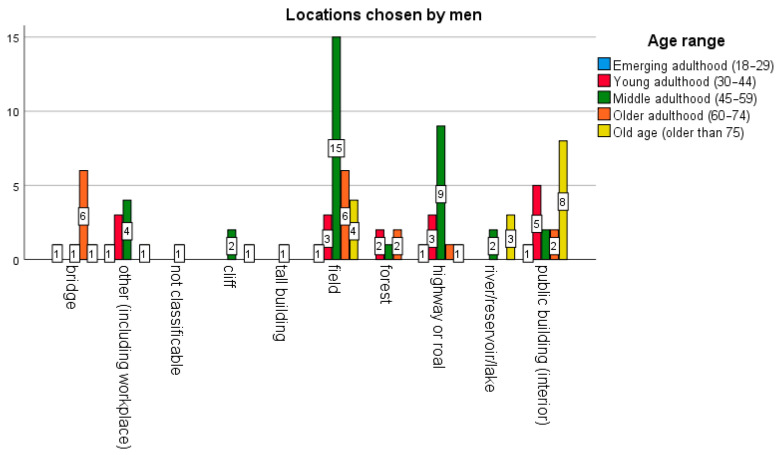
Number of cases involving men (n = 97) at public sites classifiable as critical. Analysis by age range and method.

**Figure 4:**
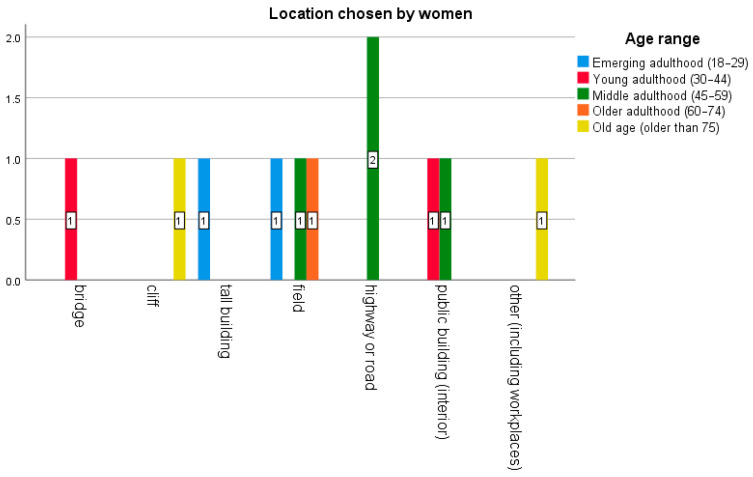
Number of cases involving women (n = 11) at sites classified as critical. Analysis by age range and method.

In terms of location (Figure 3 and Figure 4), men died by suicide more frequently in rural or transit areas (fields, highways, roads) and public buildings, while women did not choose locations such as forests or rivers. Specifically, in the case of men, the locations chosen were countryside (29 cases), highway or road (15), public building (18), bridge (9), forest (5), river or lake (5). In contrast, women made a wider variety of choices (while avoiding forests and rivers): countryside (3), road (2), public building (2), bridge (1), cliff (1), tall building (1), and other (1).

## 4. Discussion

The main objective of this study was to identify and characterize suicide locations in the provinces of Toledo and Albacete from 2020 through 2024, with the aim of identifying possible critical sites. Suicides in public places may be more easily preventable than those occurring at home, due to greater potential for observation and intervention. In addition, sociodemographic and contextual characteristics were analyzed, mainly age, sex, date, and method used, in order to generate empirical evidence that contributes to guiding prevention strategies adapted to the regional context of Castilla-La Mancha. Analysis of the sample in this study, with 421 cases, confirmed the existence of differential patterns based on sex, location, method, and seasonality. 

In terms of gender, there was a clear male predominance (82%), with a male/female ratio of 5:1, which exceeds the most prevalent ratios of 3:1, not only in Spain, but also in the European context and in high-income countries in general (GBD 2021 Suicide Collaborators, 2025; World Health Organization, 2025). This finding could be related to specific sociocultural and economic factors in the region, such as greater access to lethal or more violent methods among men, which would make them more vulnerable to dying by suicide, or the persistence of traditional gender roles that make it difficult for men to express emotional vulnerability and seek help (Simion & Jung, 2025).

With regard to age, the average age of deceased persons was 56.6 years, with a wide range from 15 to 97 years. The most highly represented age group, in raw numbers, corresponds to middle adulthood (45–59 years), followed by older adulthood (60–74 years). This pattern coincides with the national trend, where suicide mortality peaks between the ages of 45 and 60, especially among men (National Institute of Statistics [INE], 2024; World Health Organization, 2025). At these ages, vulnerability factors such as unemployment, chronic illness, social isolation, and loss of family and work roles converge (Zalsman et al., 2016). However, when age-adjusted suicide rates are considered instead of crude (absolute) numbers, international studies indicate that the highest rates are found in the population over 70 years of age (GBD 2021 Suicide Collaborators, 2025). Taken together, the results point to the need to design specific prevention strategies for middle-aged and older men in rural or semi-urban contexts, a group with high vulnerability due to a combination of personal and structural risk factors that increase the lethality of suicide attempts (Nevarez-Flores et al., 2025; Shin et al., 2025).

Of the methods used, hanging/strangulation/suffocation was chosen most frequently (56.1%), followed by jumping from a height (16.6%) and poisoning by drugs or chemicals (9.7%), which is consistent with previous studies conducted in similar contexts (GBD 2021 Suicide Collaborators, 2025). Most locations where suicides occurred were homes and private spaces, which cannot be classified as hotspots. As for other locations, a higher concentration of cases was observed in rural and semi-urban areas, especially in Toledo. In Albacete, no cases were recorded on railway tracks. The geographical configuration of Castilla-La Mancha, characterized by large rural expanses and a scarcity of urban infrastructure such as high-rise buildings, favors the occurrence of suicides on bridges and viaducts and in reservoirs and rivers, highlighting the need for adapted preventive measures such as barriers, surveillance in isolated spaces, and signage at critical sites. In any case, the figures highlight the importance of the home and private sphere as the predominant setting for recorded suicides.

The analysis of public places identified a single critical site, located in Toledo, on a high bridge associated with notable symbolism and recognized by the local population, where the main method of suicide is jumping. The interview with the forensic professional overseeing the study indicated that, due to structural and financial constraints and the extensive size of the area, the installation of full barriers may not be feasible. Nonetheless, effective alternatives exist, such as deterrent signage or the provision of helpline phones. Indeed, various authors point out that preventive measures need not completely prevent access to lethal means as partial barriers can also have a deterrent effect (Salgado, 2015). 

To the best of our knowledge, this is the first study in Spain to apply a systematic, location-based methodology to identify high-risk suicide locations in public settings, using data from two provinces as a foundation for future nationwide research. 

Based on the concentrations of cases in elevated infrastructure and public access spaces, the authors of this study recommend that the installation of physical barriers be considered a priority strategy for suicide prevention that cannot be postponed, as there is strong evidence that restricting access to hazardous sites is associated with a decrease in suicide and is therefore a key strategy that should be integrated into various public policies (Zalsman et al., 2016). The installation of such barriers should be accompanied by complementary interventions, especially in contexts where complete barrier installation is not feasible. Complementary interventions that can maximize the preventive impact include signage with help resources, crisis hotlines, and/or surveillance systems (Pirkis et al., 2015). Although initial cost and esthetic objections are often presented as obstacles, the benefits in terms of reducing deaths (over 90% in many cases) amply justify their implementation and maintenance as a public health measure. 

With regard to recorded data on suicide, various authors highlight the importance of forensic sources in improving the quality of information and reducing the underestimation of cases (Barbería et al., 2018; Giner & Guija, 2014; Santurtún et al., 2016). In Spain, the National Statistics Institute (INE), under a collaboration agreement established for this purpose with the Institutes of Legal Medicine and Forensic Sciences (IMLCF) to directly incorporate causes of death from judicial sources, provides detailed annual information on deaths by suicide, including variables such as sex, age, place of residence, month of death, and the suicide method used. This agreement arose from the need to improve data on judicial deaths which, although representing a small percentage of total deaths, include certain causes that are preventable and affect all age groups, such as completed suicides.

This study has some limitations. First, the data analyzed include only completed suicides, excluding non-fatal attempts recorded by emergency or health services, which limits overall understanding of the problem. It would be advisable to promote the integration of data from the IMLCF records with other relevant sources (emergency services, hospital records, mental health professionals, police forces) in order to obtain a more complete picture that includes both completed suicides and non-fatal attempts. In general, establishing channels for inter-institutional collaboration (involving, e.g., health, justice, social services, or education) would facilitate the implementation of prevention policies based on scientific evidence.

Second, the available information is derived from administrative records whose completeness may vary depending on the quality of reporting and on the coding process, which may introduce biases. For the purpose of conducting more robust interprovincial comparative studies and extrapolations, it would be appropriate to propose that, in the case of records of completed suicides compiled by the Institutes of Legal Medicine and Forensic Sciences and provided to the National Statistics Institute (INE), the variable “place of occurrence” be incorporated in addition to the current variables of sex, age, date, and method of death. This inclusion would be consistent with the data already collected for natural deaths documented by forensic physicians (e.g., public space, residence, workplace, among others), with the aim of identifying critical points or areas of higher incidence (Consejo Médico Forense, 2024). 

For future research, it will be essential to extend the analysis to all five provinces of Castilla-La Mancha and to adopt longer observation periods, as this would provide a more complete regional overview and allow meaningful comparisons across territories. The present study was limited to the provinces of Toledo and Albacete due to differences in forensic and healthcare data registration systems, a constraint that underscores the need for greater harmonization of data collection procedures across institutions. Addressing these disparities would reduce selection bias and improve the generalizability of future findings. At the same time, information on non-fatal suicide attempts recorded by emergency and health services should be incorporated into future studies to provide a more comprehensive approach to the phenomenon of suicide. It would also be relevant to incorporate contextual and socioeconomic variables, such as unemployment, education level, rural or urban setting, and accessibility of mental health services, with the aim of exploring possible associations with the critical sites identified, in line with other international studies. Another aspect that has been little researched relates to the impact of the media on the amplification of suicide, especially at these critical sites, and the potential for managing that impact as a preventive measure (García-Fernández et al., 2024). Also under-researched is the influence of urban design, including the creation of green spaces and facilitators of interpersonal relationships (Barboza-Salerno et al., 2025; Shen et al., 2022). Finally, the development of longitudinal studies will permit analysis of the temporal evolution of these concentrations and a more accurate assessment of the impact of any preventive measures implemented. 

## Figures and Tables

**Table 1:** Cross-tabulation by province of the variables *location* and *method* (Toledo = 271; Albacete = 150).

Method										
Location	Jumping	Hanging	Firearm	Poisoning	Inhalation	Drowning	Stabbing	Vehicle Collision	Total	Total
									Toledo/Albacete	
Bridge	5/4	1/0	0/0	0/0	0/0	0/0	0/0	0/0	6/4	10
Cliff	1/3	0/0	0/0	0/0	0/0	0/0	0/0	0/0	1/3	4
High-rise building	12	0/0	0/0	0/0	0/0	0/0	0/0	0/0	12/0	12
Field	1/0	12/14	1/2	1/0	1/0	0/0	0/0	6/0	22/16	38
Forest	0/0	1/1	2/1	0/0	0/0	0/0	0/0	0/0	3/2	5
Highway or road	3/0	5/2	2	1/0	0/0	0/0	1/0	3/1	15/3	18
River, reservoir	0/0	0/0	0/0	0/0	0/0	5/0	0/0	0/0	5/0	5
Public building (interior)	5/1	7/1	1/1	3/0	0/0	0/0	1/0	0/0	17/3	20
Other	0/0	9/3	0/0	1/0	0/0	0/1	1/0	0/0	11/4	15
Not classifiable as critical site	6/29	125/55	15/9	21/14	3/2	0/3	9/3	0/0	179/115	294
Totals	33/37	160/76	21/13	27/14	4/2	5/4	12/4	9/1	271/150	421

Note: The absolute numbers of cases per cell are indicated.

## Data Availability

The data that support the findings of this study are available from the corresponding author upon reasonable request, in compliance with ethical guidelines and participant confidentiality.
